# Normative Data for In-Hand Manipulation Skill Test for Children: A Study From Southern India

**DOI:** 10.7759/cureus.77841

**Published:** 2025-01-22

**Authors:** Rahiba C K, Renukadevi Mahadevan, Vijay S Raj V

**Affiliations:** 1 Department of Musculoskeletal, JSS College of Physiotherapy, Mysore, IND; 2 Department of Cardiorespiratory Sciences, JSS College of Physiotherapy, Mysore, IND; 3 Department of Sports Sciences, JSS College of Physiotherapy, Mysore, IND

**Keywords:** children, hand function, in-hand manipulation, normative data, tool

## Abstract

Background: In-hand manipulation skills (IHMS) are components of fine motor skills that are routinely used by children and adults during activities of daily living, recreation, and work. The only readily available tool to comprehensively assess in-hand manipulation (IHM) in children between the ages of three and nine is the test of in-hand manipulation (TIHM). However, like any norm-referenced test, TIHM requires normative values for accurate interpretation. Unfortunately, normative data for TIHM is not currently available.

Objective: The objective is to develop normative values of TIHM in typical children between three and nine years of age in Kerala.

Methodology: This study included 793 children. The inclusion criteria included girls and boys between three and nine years of age. Cluster sampling was used for data collection. The whole activity sequence was videotaped and analyzed for manipulation skills, and scoring was done. Data was analyzed using IBM SPSS Statistics for Windows, Version 22 (Released 2013; IBM Corp., Armonk, New York, United States).

Result: Most of the children from six to eight years of age scored the maximum range for all the components. Most of the children between three and four years of age used more than one compensatory method rather than manipulation, and they had difficulty following instructions and they took more time. There is no significant difference between the performance of girls and boys of the same age group in any of the variables observed.

Conclusion: Normative values of TIHM in children between five and nine years have developed.

## Introduction

The hand is a complex structure composed of numerous bones, muscles, and ligaments that facilitate various movements. These movements can be categorized into gross movements such as grasping and releasing, prehensile movements, and the intricate movements involved in in-hand manipulation (IHM). In-hand manipulation skills (IHMS) are integral to fine motor skills and crucial for everyday activities, recreation, and work among both children and adults. IHMS refer to the ability to adjust and position objects within the hand without requiring the assistance of the other hand. Exner defined IHMS as the "adjustment of an object within the hand for optimal orientation after grasp" and classified these skills into three types: translation, shift, and rotation [1]. According to Versveld, translation involves coordinated movement of the thumb and index finger, or the thumb, index, and middle fingers, in specific patterns either toward or away from the palm. Two types of translation are recognized: finger-to-palm translation, where an object moves from the finger pads to the palm, and palm-to-finger translation, where an object moves from the palm to the finger pads [2]. Shift refers to the adjustment of an object by alternating movement between the finger and thumb pads, while rotation involves moving an object around one or more of its axes. These skills can be performed while holding one or more objects within the palm. Shift and rotation can further be classified into simple and complex maneuvers.

IHMS are fine motor abilities used regularly by children and adults in daily activities, leisure pursuits, and professional tasks. These skills are influenced by motor skills, motor planning, cognitive function, and perceptual understanding [1]. It is possible that the level of IHMS could affect the ability of typically developing children to perform everyday tasks [2]. Children experiencing difficulties with IHMS may exhibit clumsiness when handling objects, demonstrate slower completion of tasks, and achieve lower academic performance. There are limited tests and protocols to assess the IHMS reported in the literature, namely, the test of in-hand manipulation (TIHM) [3]. Each of these tests has advantages and disadvantages over the other. When a therapist observes the hand manipulation skills in a child, it helps them better understand the child's fine motor abilities. It's important to consider the differences in how children perform when planning evaluations and treatments [4,5]. Therefore, assessing these skills should involve various tasks that encompass all these dimensions. A child's proficiency in IHM is linked to their motor skills, perceptual understanding, motor planning, and cognitive function [6]. Utilizing a diverse range of materials and objects can aid in developing the child's grasp of perceptual aspects related to manipulation skills. For instance, using a pencil might be more effective than a plastic peg toy in enhancing the learning experience.

The only readily available tool to comprehensively evaluate IHM in children is the norm-referenced TIHM. It can be used as an outcome measure in children with various hand dysfunctions [7]. Any norm-referenced test requires culture- and age-specific normative values for interpretation. Normative values of TIHM are not available. Hence, the study's objective was to develop normative values of TIHM in typical children from Kerala between three and nine years of age. No published evidence exists about the normative values of TIHM in children. Since it is a norm-referenced tool, normative values must be developed. Our center caters to many children from Kerala, so we need normative values from this state. Hand manipulation has been reported to change with exposure to higher-skilled activities related to cultural practices, so there is a need to develop norms based on the child's cultural context.

Clinical significance

The development of normative values will make the tool usable in a clinical situation to interpret skill development.

## Materials and methods

This study involved school-going children and permission from the relevant authorities. For the standardization of the study procedure, two specific districts from the states of Karnataka and Kerala were chosen for their convenience after complete scrutiny of the advantages and disadvantages. Permission was obtained from the Deputy Director of Public Instruction in Mysore and Kerala for this purpose. The sampling sources for the study included playschools, nurseries, and selected schools in Kerala. Ethical approval was obtained from the institutional ethical committee.

The study was conducted in two phases: phase 1, standardization; and phase 2, main study. The phase 1 study was carried out to standardize the procedure including the methods of different groups of children and materials modifications. The feasibility of the study also was analyzed, and suitable changes were carried out. Girls and boys between three and nine years of age whose development is typical as per the mother's report were included. Children with hand fractures, pain while performing activities, children with developmental delay and cognitive dysfunctions, and children who were not able to understand and follow commands were excluded from the study. Signed informed consent was received from the head of a particular institution and the parents of the children. Verbal assents were taken from the participants immediately preceding the testing.

Phase 1: standardization of the procedure of the study

**Figure 1:**
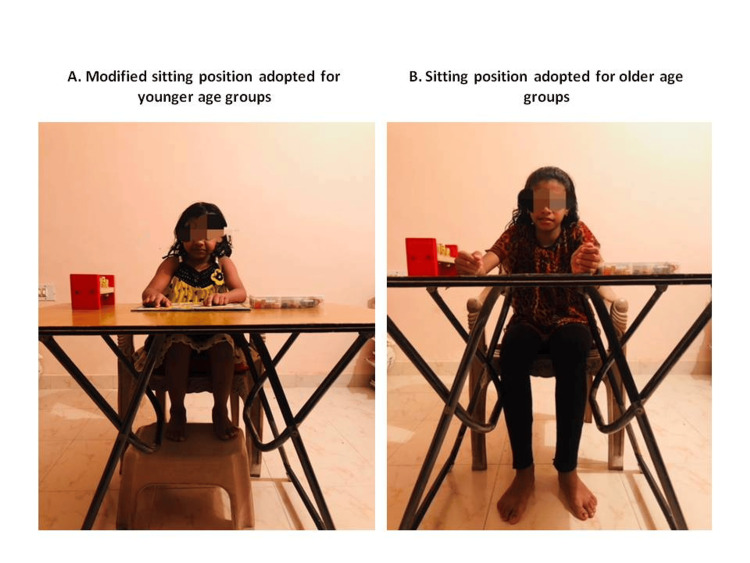
Position during the TIHM evaluation TIHM: test of in-hand manipulation

Twenty school-going children from the Mysore district were selected for convenience. A chair with a writing pad attached was used, where children were asked to be seated with their femur fully supported and feet in plantigrade on a firm surface and the writing pad adjusted to approximately at or just above the child’s elbow height (Figure 1). Before the beginning of the assessment, a paper and pen were positioned on the desk in front of the child, and he/she was asked to write his/her name. The hand naturally used by the child without prompting was recorded as dominant. Verbal commands were given throughout the tasks mentioned under TIHM to get the tasks done. A video camera was set up in front of the child in a minimally distracting location, and recordings were then used for scoring. The scores were then analyzed for consistency, and the procedure was subjectively analyzed for feasibility, accuracy, and the need for alterations. Based on the findings, the positioning and scoring pattern of the procedure was modified and standardized, which was used in the data collection.

Phase 2: participant selection, data collection and analysis

A lottery method was used to select children from five to nine years of age. Kozhikode District out of the 14 districts in Kerala was selected based on thorough analysis and feasibility. To minimize bias, one district was randomly selected using the lottery method for the sample. The sampling frame of this observational study included playschools, nurseries, and selected schools. The two subcategories made among the four and three age groups were children attending nursery schools and not attending nursery schools. In this sample source, most of the children in age group three did not attend play schools.

Cluster sampling of children from five to nine years of age and snowball sampling for children three and four years of age were used to achieve the estimated required sample size of 900 with equal distribution in age and gender groups.

Data collection procedure

For children between five and nine years of age, the time to collect the data was fixed by the class teacher. Chairs and tables that are available at the school were used. The children were asked to be seated, with their elbows at approximately a 90-degree angle to the table. Each student was explained about each task and asked to perform the task. The procedures used were standardized and as mentioned in phase 1. The whole sequence was videotaped, analyzed, and scored. For children between three and four years of age, each task was demonstrated. Then, the child was encouraged to perform the task. The whole sequence was videotaped. Descriptive data analysis was done using IBM SPSS Statistics for Windows, Version 22 (Released 2013; IBM Corp., Armonk, New York, United States), and range, mean, standard deviation (SD), median, and interquartile range (IQR) scores for each activity were computed for each age group and gender separately. 

## Results

**Figure 2:**
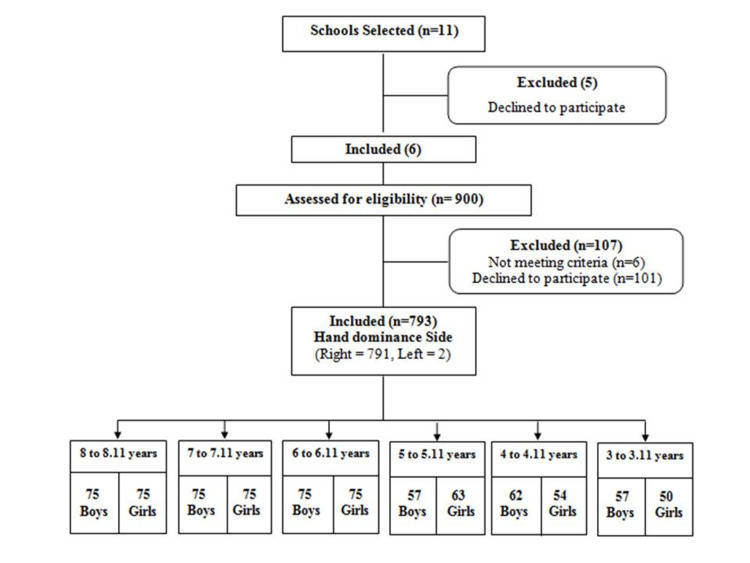
Flow process for the study participants

The present study included 900 participants, out of whom 793 children completed the study (Figure 2). Concerning hand preference, only two children were left-handed.

The descriptive data (Table 1) indicate the number of boys and girls of different age groups who completed the study.

**Table 1:** Number of boys and girls with different age group who participated in the study Values in numbers

Age (years)	3 to 3.11	4 to 4.11	5 to 5.11	6 to 6.11	7 to 7.11	8 to 8.11
Boys (N)	57	62	57	75	75	75
Girls (N)	50	54	63	75	75	75

The TIHM contains 47 activities with different IHMS, including finger-to-palm translation, palm-to-finger translation, shift, simple rotation, and complex rotation. The following tables indicate the scores for each function of IHM at various hand functions.

The normative values of translation components among children of respective age groups and genders are depicted concerning the children attending school and not attending school (Table 2). The normative range indicates the lower and upper limits. When utilizing the TIHM, it is advisable to compare TIHM scores to normative values as shown in the appendices for clearer interpretation.

**Table 2:** Scores for translation with and without stabilization in children between three and nine years of age (N = 793) attending school and not attending school SD: standard deviation; IQR: interquartile range

Age (years)	Gender	Children attending school			
		Range	Mean (SD)	Median	IQR (25-75)
8 to 8.11	Boy	54	54.00 (0)	54.00	50-54
8 to 8.11	Girl	54	53.92 (0.27)	54.00	50-54
7 to 7.11	Boy	53-54	53.97 (0.16)	54.00	50-54
7 to 7.11	Girl	53-54	53.93 (2.97)	54.00	50-54
6 to 6.11	Boy	47-50	48.64 (2.95)	48.00	50-48
6 to 6.11	Girl	46-51	49.01(2.89)	42.00	50-48
5 to 5.11	Boy	37-39	38.22 (1.24)	38.00	50-38
5 to 5.11	Girl	37-39	38.82 (1.99)	38.00	50-38
4 to 4.11	Boy	25-35	26.43 (9.14)	26.00	50-26
4 to 4.11	Girl	25-33	25.87 (10.84)	25.00	50-25
3 to 3.11	Boy	21-34	23.85(4.12)	21.00	50-21
3 to 3.11	Girl	21-31	23.85 (4.12)	21.00	50-21
Children not attending school					
4 to 4.11	Boy	21-26	21.52 (4.89)	21.00	50-21
4 to 4.11	Girl	21-28	22.48 (3.63)	22.00	50-22
3 to 3.11	Boy	16-18	16.57 (0.75)	16.00	50-16
3 to 3.11	Girl	10-7	13.89 (2.99)	15.00	50-15

The normative values of shift components among children of respective age groups and gender are shown in Table 3. The normative range indicates the lower and upper limits. When utilizing TIHM, it is advisable to compare TIHM scores to normative values (Appendices Attachment 1) for clearer interpretation.

**Table 3:** Scores for shift with and without stabilization in children between three and nine years of age (N = 793) attending school and not attending school

Age (years)	Gender	Range	Mean (SD)	Median	IQR
Children attending school					
8 to 8.11	Boy	15	15 (0)	15	50-15
8 to 8.11	Girl	15	15 (0)	15	50-15
7 to 7.11	Boy	15	15 (0)	15	50-15
7 to 7.11	Girl	15	15 (0)	15	50-15
6 to 6.11	Boy	13-15	14.10 (0.63)	14	50-14
6 to 6.11	Girl	13-15	14.19 (0.65)	14	50-14
5 to 5.11	Boy	9-11	9.07 (1.36)	9	50-9
5 to 5.11	Girl	9-10	8.84(2.04)	9	50-9
4 to 4.11	Boy	5-7	5.43 (1.86)	5	50-5
4 to 4.11	Girl	5-7	4.72 (2.42)	5	50-5
3 to 3.11	Boy	3-7	5.75 (1.29)	5	50-7
3 to 3.11	Girl	5-7	6.14 (1.01)	7	50-7
Children not attending school					
4 to 4.11	Boy	5-7	5.86 (1.83)	5	50-5
4 to 4.11	Girl	5-7	4.87 (2.54)	5	50-5
3 to 3.11	Boy	2-5	3.05 (1.20)	3	50-3
3 to 3.11	Girl	2-5	3.61 (1.33)	3	50-3

The normative values of simple rotation components among children of respective age groups and genders are shown in Table 4. The normative range indicates the lower and upper limits. When utilizing TIHM, it is advisable to compare TIHM scores to normative values as shown in the appendices for clearer interpretation.

**Table 4:** Scores for simple rotation with and without stabilization in children between three and nine years of age (N = 793) attending school and not attending school SD: standard deviation; IQR: interquartile range

Age (years)	Gender	Range	Mean (SD)	Median	IQR
Children attending school					
8 to 8.11	Boy	34-36	35.79 (0.62)	36.00	50-36
8 to 8.11	Girl	34-36	35.60 (0.75)	36.00	50-36
7 to 7.11	Boy	33-36	35.93 (0.25)	36.00	50-36
7 to 7.11	Girl	33-36	35.56 (0.66)	36.00	50-36
6 to 6.11	Boy	29-35	32.75 (2.63)	34.00	50-34
6 to 6.11	Girl	29-36	33.00 (2.28)	34.00	50-34
5 to 5.11	Boy	18-20	18.70 (4.66)	19.00	50-18
5 to 5.11	Girl	17-18	16.75 (4.37)	18.00	50-19
4 to 4.11	Boy	14-15	13.73 (3.66)	15.00	50-15
4 to 4.11	Girl	14-15	11.89 (5.74)	14.00	50-14
3 to 3.11	Boy	7-13	9.00 (1.88)	10.00	50-10
3 to 3.11	Boy	7-11	10.75 (1.42)	10.00	50-10
Children not attending school					
4 to 4.11	Boy	5-11	7.12 (3.51)	10.00	50-10
4 to 4.	Girl	5-11	6.98 (4.23)	10.00	50-10
3 to 3.11	Boy	2-10	3.16 (1.50)	2.00	50-2
3 to 3.11	Girl	2-5	4.2857 (1.20)	2.00	50-2

The normative values of complex rotation components among children of respective age groups and genders are shown in Table 5. The normative range indicates the lower and upper limits.

**Table 5:** Scores for complex rotation with and without stabilization in children between three and nine years of age attending school and not attending school SD: standard deviation; IQR: interquartile range

Age (years)	Gender	Range	Mean (SD)	Median	IQR
Children attending school					
8 to 8.11	Boy	35-36	35.65 (0.47)	36.00	50-36
8 to 8.11	Girl	34-36	35.69 (0.46)	36.00	50-36
7 to 7.11	Boy	34-36	35.56 (0.73)	36.00	50-36
7 to 7.11	Girl	34-36	35.64 (0.51)	36.00	50-36
6 to 6.11	Boy	30-33	31.42 (1.36)	31.00	50-31
6 to 6.11	Girl	29-33	31.22 (1.67)	31.00	50-31
5 to 5.11	Boy	20-28	24.66 (4.54)	27.00	50-27
5 to 5.11	Girl	20 -24	21.49 (5.05)	23.00	50-23
4 to 4.11	Boy	9-12	9.62 (3.86)	9.00	50-9
4 to 4.11	Girl	9-12	9.27 (3.15)	9.00	50-9
3 to 311	Boy	7-9	7.47 (0.87)	9.00	50-7
3 to 3.11	Girl	7-9	8.08 (1.01)	7.00	50-9
Children not attending school					
4 to 4.11	Boy	3-9	6. 21 (2.50)	6.00	50-6
4 to 4.11	Girl	3-9	6.78 (2.99)	6.00	50-6
3 to 3.11	Boy	3-5	4.16 (0.92)	3.00	50-5
3 to 3.11	Girl	3-5	3.28 (0.71)	3.00	50-3

When utilizing TIHM, it is advisable to compare TIHM scores to normative values as shown in the appendices for clearer interpretation. The normative value table enhances objectivity and precision in evaluating and understanding individual performance or outcomes.

## Discussion

To establish normative values for children, it is recommended to include between 50 and 75 participants in each age group [8]. This study enrolled over 50 participants per age group. Children aged six, seven, and eight years demonstrated proficient performance in all translation activities without needing compensation. Five-year-olds efficiently performed finger-to-palm translation but required more time for palm-to-finger translation activities. In contrast, children aged three and four years took significantly longer, displayed clumsiness, and employed various compensatory techniques. These findings align with Exner's observations that translation skills begin rapid development around age one, with five-year-olds typically mastering finger-to-palm and seven-year-olds mastering palm-to-finger translation [9].

Older children, specifically those aged six, seven, and eight years, executed shift tasks without compensatory methods, suggesting mastery of shift components of IHM by age six. Effective shift IHM demands stable wrist and forearm control, intrinsic muscle movements, and a stable opposing thumb [9]. These components tend to be more developed in older children, facilitating precise object manipulation. Younger children (ages three to five years) relied on compensatory methods and took longer to complete tasks. Rotation skills begin development around age three and are generally mastered by age five. Most eight- and seven-year-olds completed rotation tasks without compensatory methods, while five- to six-year-olds used compensatory techniques. Common compensatory methods included rotating the arm or forearm, with younger children often rotating the entire hand instead of the object, resulting in more drops. Children aged three to four years primarily used their hands for grasp without noticeable IHM. Most eight- and seven-year-olds performed tasks involving IHM without difficulty and achieved higher scores compared to younger and older groups. Biomechanical hand structures and related motor skills are more developed in older age groups, contributing to their performance advantages. Children aged three to four years generally scored lower, potentially due to difficulties understanding instructions. It was observed in the present study that children aged three to four took more time to understand the given instruction which resulted in less score compared to the other age groups, which is also supported by the study conducted in children aged 4-7, by Stone et al. [10].

The development of IHM skills typically begins around 12 months and progresses rapidly from ages three to six [11]. While it is commonly believed that five-year-olds master hand manipulation skills, this study indicates that although most five-year-olds exhibit basic skills, they often lack precision in task execution. Children aged three to four years often prematurely release objects after grasping, except for those in school at age four who exhibit better task execution abilities. Compensatory methods observed included stabilization against the body or table, wrist rotation, and using both hands or switching hands, with younger children (ages three to four) often employing multiple compensatory movements. As age increases, the time required to complete IHM tasks decreases, and the consistency and maturity of manipulation techniques improve. This trend is consistent with observations of eight-year-olds manipulating objects against gravity [12,13]. Subgroup analysis between children attending nursery and those not attending school revealed significantly lower scores among non-school attendees, likely due to less exposure to craft activities that promote hand manipulation skills [14]. Motor skills, including muscle strength, control, perceptual understanding, motor planning, and cognitive function, collectively contribute to IHM skill development [14,15]. Children aged three to four years often struggled with task execution due to difficulty following instructions and prolonged completion times [16], suggesting that TIHM assessments may not be suitable for this age group. TIHM assessments are more beneficial for children over five years who demonstrate better task understanding and execution based on instructions.

The strength of the study

This study established standard scores for TIMS in children aged five to nine years, including the time required to complete each component. These norms can be utilized in clinical settings to assess hand manipulation skill development and as a measurement of the treatment protocol's effectiveness.

Limitation and future implications

This study's limitation is that it considered children from one district of India. IHM is dependent on experience and cultural factors. Hence, these norms may not be relevant to other groups of children. A more extensive study with sampling from India would be required to overcome this limitation.

## Conclusions

TIHM is a convenient and thorough assessment tool designed to evaluate children's IHMS. It provides standardized measurements and is widely available for use. Normative values for TIHM have been established specifically for children aged five to nine from the Kozhikode District of Kerala.

## Appendices

**Table 6:** Normative reference values of TIHM in children between five and nine years of age TIHM: test of in-hand manipulation

Age (year)	Sex	Translation	The time required to complete translation (seconds)	shift	The time required to complete a shift (seconds)	Simple rotation	The time required to complete simple rotation (seconds)	Complex rotation	The time required to complete complex rotation (seconds)
8-8.11	Boy	54	56.32-59.65	15	19.32-21.96	34-36	60.45-63.98	35-36	45-47.65
8-8.11	Girl	54	56.01-58.77	15	19.18-21.68	34-36	60.01-63.65	34-36	45.23-48.78
7-7.11	Boy	53-54	55.45-64.16	15	21.23-27.31	33-36	65.21-71.79	34-36	48.31-59.74
7-7.11	Girl	53-54	55.45-62.78	15	21.23-25.67	33-36	65.12-70.81	34-36	50.07-51.58
6-6.11	Boy	47-50	66.45-79.67	13-15	30.45-40.71	29-35	69.45-80.83	30-33	60.46-70.62
6-6.11	Girl	46-51	68.51-80.07	13-15	29.75-42.27	29-36	71.45-82.8	29-33	63.31-72.12
5-5.11	Boy	33-39	79.67-104.05	9-11	35.39-41.34	18-20	82.07-109.67	20-28	78.15-96.78
5-5.11	Girl	35-39	76.84-99.51	9 -10	35.74-43.09	17-18	86.31-109.32	20-24	79.56-101.32
